# Glutamate enhances the production of inflammatory cytokines IL-6 and IL-11, as well as chemokines CXCL2, CXCL3, and CXCL8 in keloid fibroblasts

**DOI:** 10.3389/fmolb.2025.1720876

**Published:** 2026-01-05

**Authors:** Yan Chen, Yaohan Xu, Jiahe Zhang, Chenxi Feng, Jie Chen, Yinjing Song, Jing Pan, Jiang Zhu, Hao Cheng

**Affiliations:** 1 Department of Dermatology and Venereology, Sir Run Run Shaw Hospital, Zhejiang University School of Medicine, Hangzhou, China; 2 Center for Plastic and Reconstructive Surgery, Zhejiang Provincial People’s Hospital, Affiliated People’s Hospital, Hangzhou Medical College, Hangzhou, China

**Keywords:** fibroblast, glutamate, GRIN2D, inflammation, keloid

## Abstract

**Introduction:**

Keloids are fibroproliferative skin scars characterized by excessive extracellular matrix deposition and a high rate of recurrence. Despite extensive research, their pathogenesis remains incompletely understood and effective curative therapies are lacking.

**Methods:**

RNA sequencing (RNA-seq) and metabolomics were performed to compare gene expression and metabolite profiles between human keloid tissues and normal skin. Single-cell RNA sequencing, immunohistochemistry, and immunofluorescence were used to determine the cellular localization of key genes. *In vitro*, human fibroblasts were stimulated with glutamate, followed by RNA-seq, quantitative RT-PCR, and ELISA to evaluate inflammatory gene expression and cytokine secretion.

**Results:**

Transcriptomic analysis revealed significant enrichment of the neuroactive ligand-receptor interaction pathway in keloid tissue, with marked upregulation of the glutamate receptor subunit GRIN2D. Single-cell and histological analyses demonstrated that GRIN2D is predominantly expressed in fibroblasts. Metabolomic profiling showed significantly increased levels of glutamate and glutamine in keloid tissues. Glutamate stimulation of fibroblasts significantly enhanced the expression and secretion of inflammatory cytokines IL-6 and IL-11, as well as chemokines CXCL2, CXCL3, and CXCL8 (IL-8).

**Discussion:**

These results underscore the crucial role of glutamate metabolism in promoting the infammatory functions of fbroblasts. They suggest that glutamate contributes to keloid progression and provides a theoretical basis for targeting glutamte signaling pathway in keloid treatment.

## 1 Introduction

Keloids are benign fibroproliferative dermal tumors that arise from skin trauma. They are characterized by excessive accumulation of extracellular matrix (ECM), especially collagen, in the dermis and subcutaneous tissue, and they extend beyond the margins of the original wound (Bran et al., 2009; Limandjaja et al., 2020). Keloids can cause severe pain, cosmetic harm, pruritus and movement restriction due to their scar-like character (Balci et al., 2009; Direder et al., 2022; Ud-Din and Bayat, 2014). Genetic susceptibility and chronic inflammatory processes have been implicated in their etiology (Alhady and Sivanantharajah, 1969; Kiprono et al., 2015; Ramakrishnan et al., 1974), although the exact causes of keloid scarring remain unknown. Several treatment options are available, including steroid injections, γ-radiation, and surgery (Mustoe et al., 2002; Ud-Din and Bayat, 2014). However, keloids frequently recur, and recurrence often worsens the condition. Improved understanding of the pathomechanisms underlying keloid formation may help identify specific therapeutic targets and improve treatment for this currently intractable disorder.

Current studies have identified a number of factors involved in keloid development. Transforming growth factor-β (Bai et al., 2024; Li et al., 2024), vascular endothelial growth factor (VEGF) (Wu et al., 2006), and platelet-derived growth factor (PDGF) have all been shown to play important roles in collagen deposition and tissue angiogenesis (Cai et al., 2025). Additionally, heparin, fibroblast growth factors β (FGF-β), insulin-like growth factor (IGF)and IL-8 have been implicated in angiogenesis during would healing (Narayanan et al., 2024; Yoon et al., 2024). Tumor necrosis factor (TNF), IL-6, and IFN-β are upregulated in keloids, promoting cell migration and proliferation (Berman et al., 2017). Multiple fibrotic signaling cascades contribute to keloid development. The SMAD signaling pathway is a downstream mediator of TGF-β; phosphorylation of R-SMAD3 is upregulated in keloids, whereas downregulation of R-SMAD3 markedly decreases procollagen gene expression in keloid fibroblasts (Wang et al., 2007). Keloid formation can be substantially attenuated by inhibition of the TGF-β1/SMAD signaling pathway and by activation of TLR7 or SMAD7 (Xie et al., 2023; Zhang et al., 2019). Toll-like receptors (TLRs) are also key mediators in the pathogenesis of fibrosis. Following skin injury, TLRs are activated by damage-associated molecular patterns (DAMPs), enabling innate immune cells to release multiple pro-inflammatory and pro-fibrotic cytokines, which in turn enhances collagen production (Beutler, 2007; Bhattacharyya et al., 2013; Okamura et al., 2001).

Glutamate serves as a central hub in cellular metabolism and signaling (Vidor et al., 2022). At the metabolic level, it functions as a key amino group donor and links carbon and nitrogen metabolism (Wang et al., 2025). In addition, glutamate is an essential precursor for glutathione (GSH) synthesis, thereby playing a pivotal role in maintaining redox homeostasis (Kanagasabai et al., 2024). Intracellular glutamate is derived predominantly from the catabolism of glutamine and can be exported into the extracellular space via specific transporters (Mozafari et al., 2023). Once released, extracellular glutamate activates ionotropic (NMDA, AMPA, kainate) and metabotropic glutamate receptors, thereby inducing rapid cation influx—particularly Ca^2+^ or triggering G protein–mediated second-messenger cascades (Benvenutti et al., 2022). Through these pathways, glutamate couples cellular metabolic status to long-lasting intracellular signaling, gene expression programs and cell fate decisions, and contributes to both physiological processes (Chokkiveettil et al., 2025). GRIN2D (Glutamate ionotropic receptor NMDA Type subunit 2D) is a subtype of ionotropic glutamate receptors (Eyal, 2025). As a glutamate-gated Ca^2+^ permeable channel component, GRIN2D functions as a key molecular mediator linking extracellular glutamate signaling to long-lasting intracellular calcium-dependent pathways that regulate gene expression, proliferation, and cell fate (XiangWei et al., 2019).

In this study, we identified a marked upregulation of the glutamate receptor subunit GRIN2D in fibroblasts residing within keloid tissue. Functionally, *in vitro* stimulation of fibroblasts with glutamate, coupled with RNA-seq, RT–PCR, and ELISA analyses, revealed that glutamate robustly amplifies the production of inflammatory mediators, notably the cytokines IL-6 and IL-11 and the chemokines CXCL2, CXCL3, and CXCL8. Collectively, these findings indicated that fibroblast-intrinsic glutamate signaling, driven by glutamine metabolism, critically potentiates the inflammatory phenotype of keloid fibroblasts. This work therefore implicated the glutamine–glutamate–GRIN2D axis as an important contributor to keloid progression and highlights it as a potential metabolic and signaling target for therapeutic intervention.

## 2 Materials and methods

### 2.1 Sample preparation for this study

A total of 10 patients including 7 females and 3 males participated in this study at Zhejiang Provincial People’s Hospital, Affiliated People’s Hospital, Hangzhou Medical College, Hangzhou between March–June 2025. All individuals provided written informed consent prior to study enrollment. The inclusion criteria for patients with KD were as follows: participants, aged 18–35 years (mean ± SD, 24.9 ± 3.8 years; median, 26 years). Keloid tissue samples were obtained from the earlobe (6 samples), the chest (2 samples) and the navel (2 samples) during surgery. The samples were used for RNA sequencing (RNA-seq) and untargeted metabolomics detection. The inclusion criteria for the participants from double eyelid surgery in the healthy control (HC) group were aged 18–35 years (mean ± SD, 26.3 ± 4.3 years; median, 27 years). This study received approval from the Ethics Committee of Zhejiang Provincial People’s Hospital.

### 2.2 Cells and reagents

The HKF (Human keloid fibroblasts) cell line obtained from American Type Culture Collection (CRL-1762, Manassas, VA, United States), and were cultured in Dulbecco’s modified Eagle’s medium (DMEM, Life Technologies, Carlsbad, CA, United States) containing 10% fetal bovine serum (FBS; Life Technologies) at 37 °C in a 5% humidified CO_2_ incubator. Glutamate was obtained from MedChemExpress Co., Ltd. (Shanghai, China). α-SMA (14395-1-AP) and GRIN2D (27232-1-AP) were from Proteintech (Wuhan, China).

### 2.3 Cell treatment

HKF cells were carefully prepared and seeded onto a 12-well plate at a density of 2 × 10^5^ cells/mL. After seeding, the cells were allowed to adhere and incubate overnight under standard conditions. For the intervention group, cells were exposed to glutamate at two distinct concentrations (10 μM and 100 μM) in amino acid-free DMEM supplemented with 10% fetal bovine serum for 24 h, as established by prior research methodologies (Alim et al., 2021). Each group included three replicate wells.

### 2.4 Total RNA extraction, library construction and sequencing

The pathological tissue blocks removed by surgery were washed with pre-cooled normal saline. The healthy skin from the same patient was used as a normal control. The tissue was dried with absorbent paper, and then weighed and immediately immersed in liquid nitrogen. The tissue was grinded and crushed thoroughly after freezing with liquid nitrogen using a pre-cooled mortar. Then 1 mL TRIzol™ Reagent (Thermo Fisher Scientific, Cat# 15596026) were add to destroy cell membrane and lyse cell components. 200 μL chloroform was add, cell components were shaken violently for 15 s and incubated for 2 min in room temperature. Then centrifuge at 12000 rpm for 15 min under 4 °C, and supernatant were transferred into a new tube. 0.5 mL isopropyl alcohol was add, the liquid in the tube was mix gently, and incubate at room temperature for 10 min. Centrifuge at 12000 *g* for 10 min under 4 °C and discard the supernatant. 1 mL 75% ethanol was add and gently wash the precipitation. Centrifuge at 7500 g for 5 min under 4 °C and discard the supernatant. After air dried, the mRNA was dissolved in DEPC-pretreated water.

RNA quantity and integrity were first evaluated using a Qubit™ 3.0 Fluorometer (Thermo Fisher Scientific, Cat# Q33216) and an Agilent 5300 Fragment Analyzer System (Agilent Technologies, Santa Clara, CA, United States; Cat# M5310AA). Only RNA samples with high purity $\left(A_{260}/A_{280}=1.8–2.2\right)$ and an RNA integrity number (RIN) greater than 7.0 were advanced to library construction with Illumina-compatible workflows. From 2 μg of total RNA, mRNA was enriched using mRNA Capture Beads 2.0 (Yeasen Biotechnology, Cat# 12629 ES, China) through two rounds of poly(A)-based selection.

The isolated mRNA was then fragmented at 94 °C for 5 min in a magnesium-containing fragmentation buffer (Yeasen Biotechnology, Cat# 12340ES97). First-strand cDNA was synthesized by random hexamer-primed reverse transcription using SuperScript™ IV Reverse Transcriptase (Thermo Fisher Scientific). Second-strand synthesis was performed with *E. coli* DNA Polymerase I, RNase H, and a dUTP-containing nucleotide mix (Yeasen Biotechnology, Cat# 12340ES97) to enable strand-specific library preparation. The resulting double-stranded cDNA was end-repaired and A-tailed at the 3′termini with Klenow Fragment (3′→5′exo–), followed by ligation to Illumina-compatible forked adapters (IDT) carrying T-overhangs. Adapter-ligated products were amplified by PCR and subjected to size selection with Hieff NGS DNA Selection Beads (Yeasen Biotechnology, Cat# 12601ES75) to obtain libraries with an average insert size of approximately 400 ± 50 bp. Strand specificity was preserved by dUTP-based strand marking and subsequent uracil excision. Final libraries were sequenced as 2 × 150 bp paired-end reads on an Illumina NovaSeq™ X Plus platform (LC-Bio Technology, Hangzhou, China) according to the manufacturer’s instructions.

### 2.5 Differential expression genes (DEGs) analysis

DESeq2 R package (version 4.5, URL: https://bioconductor.org/packages/release/bioc/html/DESeq2.html) was used to calculate the DEGs between normal tissues and the keloid lesional tissues. Genes with the |log2 (fold change)| ≥ 1.0 and an adjusted p value <0.05 were considered to be significant different.

### 2.6 Gene ontology (GO) analysis

The GO database is a structured standard biological model constructed by Gene Ontology Consortium in 2000, which covers three aspects including cellular component, molecular function and biological process. GO enrichment analysis was conducted in the online database: http://geneontology.org/.

### 2.7 Kyoto Encyclopedia of genes and genomes (KEGG) pathway enrichment analysis

KEGG is a database resource for understanding the advanced functions and utility of biological systems, such as cells, organisms and ecosystems, from genomic and molecular level. It is a computerized representation of biological systems, consisting of the molecular building blocks of genes and proteins (genomic information) and chemicals (chemical information), which are combined with knowledge of molecular wiring diagrams of interaction, reaction and relationship networks (systematic information). It also contains disease and drug information (health information) as disturbances to biological systems. The KEGG pathway enrichment analysis was conducted on http://www.genome.jp/kegg/.

### 2.8 Gene set enrichment analysis (GSEA)

GSEA is a computational method for determining whether an *a priori* defined set of genes exhibit statistically significant, consistent differences between two biological states (Mootha et al., 2003). GSEA has been implemented as a software tool for use with microarray data and RNA-seq. GSEA reveals many biological pathways, chromosomal location and regulation. The GSEA pathway enrichment analysis was conducted on https://www.gsea-msigdb.org/gsea/index.jsp#License%20Terms.

### 2.9 Untargeted metabolome profiling

Tissues stored at −80 °C and sent to LC-Bio Technologies (Hangzhou, China) for untargeted metabolome profiling. Skin tissue samples were homogenized with grinding beads in 500 μL of prechilled extraction solvent (methanol:acetonitrile:water, 2:2:1, v/v) supplemented with deuterated internal standards. After vortexing for 30 s, the homogenates were incubated at −40 °C for 1 h to facilitate protein precipitation. Samples were then centrifuged at 12,000 rpm (RCF = 13,800 × g, rotor radius = 8.6 cm) for 15 min at 4 °C, and the resulting supernatants were transferred to clean glass vials for LC–MS analysis. Quality control (QC) samples were generated by pooling equal aliquots of supernatants from all individual biological samples.

Targeted metabolomic profiling was conducted on a Vanquish UHPLC system (Thermo Fisher Scientific) equipped with a Waters ACQUITY UPLC BEH Amide column (2.1 mm × 50 mm, 1.7 μm) and interfaced with an Orbitrap Exploris 120 mass spectrometer (Thermo Fisher Scientific). The chromatographic separation employed a binary solvent system consisting of solvent A (25 mM ammonium acetate and 25 mM ammonium hydroxide in water, pH 9.75) and solvent B (acetonitrile). The autosampler was maintained at 4 °C, and the injection volume was set to 2 μL. The Orbitrap Exploris 120 was operated under information-dependent acquisition (IDA) control using Xcalibur software (Thermo Fisher Scientific), in which the instrument continuously interrogates full-scan MS spectra and selects precursor ions for subsequent MS/MS fragmentation. Electrospray ionization (ESI) source parameters were as follows: sheath gas flow 50 Arb, auxiliary gas flow 15 Arb, capillary temperature 320 °C, full MS resolution 60,000, MS/MS resolution 15,000, stepped normalized collision energy (NCE) of 20/30/40, and spray voltage of 3.8 kV in positive mode and −3.4 kV in negative mode.

Raw LC–MS data files were converted to mzXML format using ProteoWizard and subjected to preprocessing in R with an in-house pipeline built around the XCMS package for peak detection, extraction, alignment, and integration. Metabolite identification was performed by matching MS/MS spectra against an in-house MS^2^ library, applying an annotation score cutoff of 0.3. Data pretreatment steps included peak picking, initial and secondary peak grouping, retention time correction, and annotation of isotopic peaks and adducts using XCMS. Metabolites were further annotated by comparing the exact mass-to-charge ratios (m/z) of detected features with entries in online databases such as KEGG and HMDB. All statistical analyses were conducted in R (version 4.0.0).

### 2.10 Immunohistochemistry (IHC) assay

Keloid and healthy skin specimens were fixed in 4% paraformaldehyde for a minimum of 48 h. Paraffin-embedded tissue blocks were sectioned at a uniform thickness of 5 μm using a microtome to ensure consistency across all samples. The sections were then mounted on glass slides and processed through deparaffinization and rehydration, which included sequential xylene and graded alcohol washes. Endogenous peroxidase activity was blocked with 3% H2O2 for 10 min, followed by serum blocking with 3% BSA for 30 min at room temperature. Antigen retrieval was performed using pepsin digestion. Sections were incubated overnight at 4 °C with primary antibodies targeting GRIN2D (Proteintech, 27232-1-AP), followed by incubation with secondary antibodies for 50 min at room temperature. DAB (3,3′-Diaminobenzidine) staining was applied, and nuclei were counterstained with hematoxylin for approximately 3 min. Images were captured using a Nikon Eclipse ci microscope equipped with a Nikon digital sight DS-FI2 imaging system. The average optical density was analyzed using ImageJ software.

### 2.11 Immunofluorescence (IF) assay

Keloid and healthy skin specimens were fixed in 4% paraformaldehyde and washed with PBS three times for 5–10 min each. Samples were incubated in blocking solution for 1 h at room temperature to minimize non-specific binding. Primary antibodies α-SMA (Proteintech, 14395-1-AP) and GRIN2D (Proteintech, 27232-1-AP) were diluted in blocking solution as per the manufacturer’s instructions and incubated overnight at 4 °C or for 1–2 h at room temperature. After washing with PBS three times for 5 min each to remove unbound antibodies, secondary antibodies were diluted in blocking solution and incubated with the samples for 1 h at room temperature in the dark. The samples were washed again with PBS three times for 5 min each. Finally, mounting medium containing DAPI (or another nuclear stain) was applied, and the samples were covered with a coverslip. Images were captured using an OLYMPUS CX41-32RFL microscope.

### 2.12 Real-time PCR detection

RNA was extracted from purified fibroblasts treated with glutamate or media using Trizol (ThermoFisher Scientific) and reverse transcribed with RevertAid Kit (ThermoFischer Scientific) according to the manufacturer’s instructions. The cDNA served as a template for amplification of the genes of interest. For analysis of genes expressed by fibroblasts, primers for IL-6, IL-11, CXCL2, CXCL3, and CXCL8 were used and target-gene expression was calculated using the comparative method for relative quantification upon normalization to GAPDH gene expression.

### 2.13 ELISA assay

ELISA assay was performed by using human IL-6 and IL-11 ELISA development kits (Invitrogen, EHIL11, and BMS213-2), according to manufacturers’ instructions. ELISA plates were read on a SynergyMx M5 plate reader (Molecular Devices, San Jose, CA). Concentrations of cyto-/chemokines were calculated based on serial dilutions of standards by using GraphPad Prism software (Version 9, GraphPad Software, La Jolla, CA).

### 2.14 scRNA-seq data analysis

scRNA-seq data as previously reported (Deng et al., 2021), and downloaded in the Gene Expression Omnibus (GEO) database (GSE163973). Bioinformatics analysis utilized Cell Ranger (v7.1.0) and Seurat (v4.3.0) for data processing, clustering, and differential expression analysis. Nonlinear dimensionality reduction was performed using t-distributed Stochastic Neighbor Embedding (t-SNE) implemented in Seurat v4.3.0.

### 2.15 Statistical analysis

The data were analyzed by SPSS Statistics (version: 25.0; IBM), and measurement data were expressed as main ±standard error of the mean (main ±SEM). One-way ANOVA was used for comparison between multiple groups of data. P < 0.05 was considered to be differ significantly.

## 3 Results

### 3.1 RNA-seq and GO analysis of keloids revealed the extracellular region, immunoglobulins, and the plasma membrane associated genes were significantly changed

**FIGURE 1:**
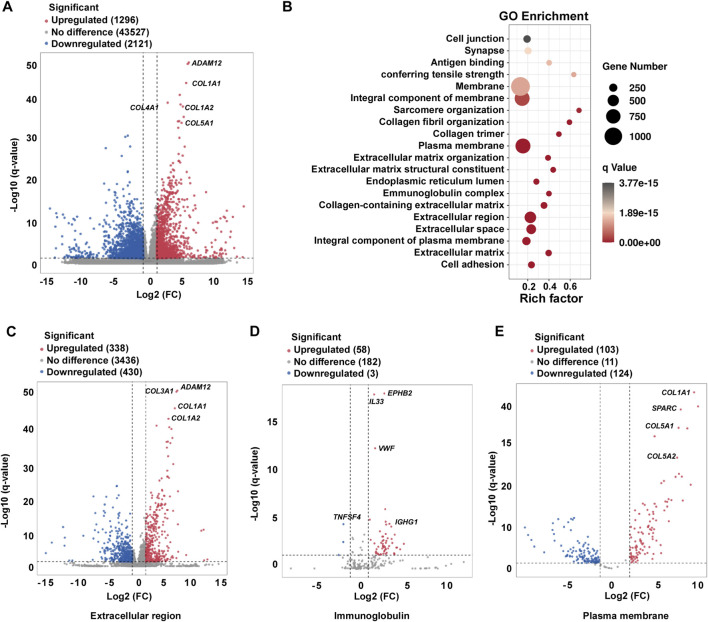
Global transcriptional alterations and GO enrichment analysis in keloid tissue. **(A)** Volcano plot illustrating the differential expression of total genes between keloid (10 samples) and normal skin controls (9 samples). **(B)** GO enrichment scatter plot showing the enriched biological processes in keloid and normal skin controls. **(C–E)** Volcano plot of genes associated with the extracellular region **(C)**, immunoglobulin-related genes **(D)**, and plasma membrane-associated genes **(E)**.

To characterize the transcriptional profiles of keloids, we performed RNA sequencing on skin tissue samples from healthy individuals and keloid patients. RNA-seq showed differentially expressed gene transcripts between keloids and health skin tissue with the transcription of 1296 genes markedly upregulated and 212 downregulated in keloid tissue (Figure 1A). In a recent study, the location of fibroblasts within the lesional skin also appears to contribute differentially to the fibrosis. Isolated deep lesional fibroblasts display elevated expressions of collagen type I alpha 1 chain (COL1A1)(Supp et al., 2012), consistent with our findings where *COL1A1*, COL1A2, *COL4A1*, and *COL5A1* were prominently upregulated in keloid tissues (Figure 1A). GO enrichment analysis indicated that pathways related to the extracellular region, immunoglobulins, and the plasma membrane were significantly enriched in keloids (Figure 1B). Specifically, half of the genes in the extracellular region pathway were upregulated, with many immunoglobulin-related genes such as *EPHB2*, *IL33*, *TNFSF4*, and *IGHG1* genes showing increased expression (Figures 1C,D). The plasma membrane-related genes were also notably upregulated (Figure 1E), highlighting the critical roles of the extracellular matrix (ECM), immune response, and membrane dynamics in keloid pathology.

### 3.2 KEGG and GSEA analysis of keloids displayed significant enrichments in the calcium signaling pathway, neuroactive ligand-receptor interaction, and ECM-receptor interaction in keloid

**FIGURE 2:**
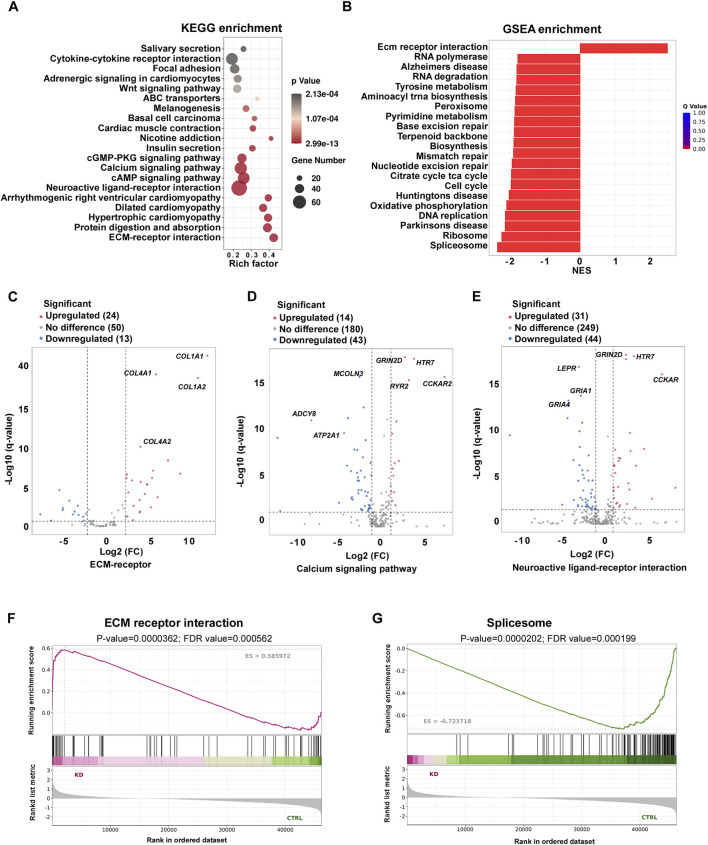
KEGG and GSEA enrichment analysis of DEGs in keloid tissue. **(A)** Scatter plot of KEGG enrichment the differential expression of total genes between keloid and normal skin controls. **(B)** Bar graph presenting the top 20 GSEA enriched pathways between keloidand normal skin controls. **(C–E)** Volcano plot analyzing genes involved in ECM-receptor interactions **(C)**, calcium signaling pathway **(D)**, neuroactive ligand-receptor interaction **(E)**. **(F,G)** GSEA of ECM-receptor interaction pathway **(F)** and splicing factor related pathway **(G)**.

To further analyze the functions of differentially expressed genes between keloid and healthy skin tissues, we conducted KEGG enrichment analysis of our RNA-seq data. The top 20 enriched pathways are depicted in Figure 2A, revealing significant enrichments in the calcium signaling pathway, neuroactive ligand-receptor interaction, and ECM-receptor interaction in keloid. Furthermore, GSEA analysis showed ECM-receptor interaction, RNA polymerase, Splicesome, and Ribosome were also significantly enriched (Figure 2B). Among the genes enriched in the extracellular region pathway, 24 genes including *COL1A1*, *COL1A2*, and *COL4A1* were significantly upregulated in keloid tissues (Figure 2C). Additionally, 14 genes such as *GRIN2D*, *HTR7*, and *CCKAR* were found to be upregulated in the calcium signaling pathway, while 43 genes, including *ATP2A1*, *ADCY8*, and *MCOLN3*, were significantly downregulated, suggesting that calcium signaling plays a critical role in the pathology of keloids (Figure 2D). Furthermore, the neuroactive ligand-receptor interaction pathway exhibited substantial changes, with 31 genes, including *GRIN2D*, *HTR7*, and *CCKAR* were upregulated and 44 genes downregulated in keloid tissues compared to normal skin controls (Figure 2E). Notably, the ECM-receptor interaction pathway also emerged prominently in the GSEA analysis (Figure 2F). Conversely, a downregulation of the spliceosome pathway was noted, indicating complex regulatory mechanisms involved in keloid formation (Figure 2G). Our results showed that calcium signaling pathway, neuroactive ligand-receptor interaction, and ECM-receptor interaction were significantly enriched by KEGG and GSEA analysis in keloid.

### 3.3 Glutamate metabolic and glutamate receptor (GRIN2D) was increased in keloids and fibroblasts

**FIGURE 3:**
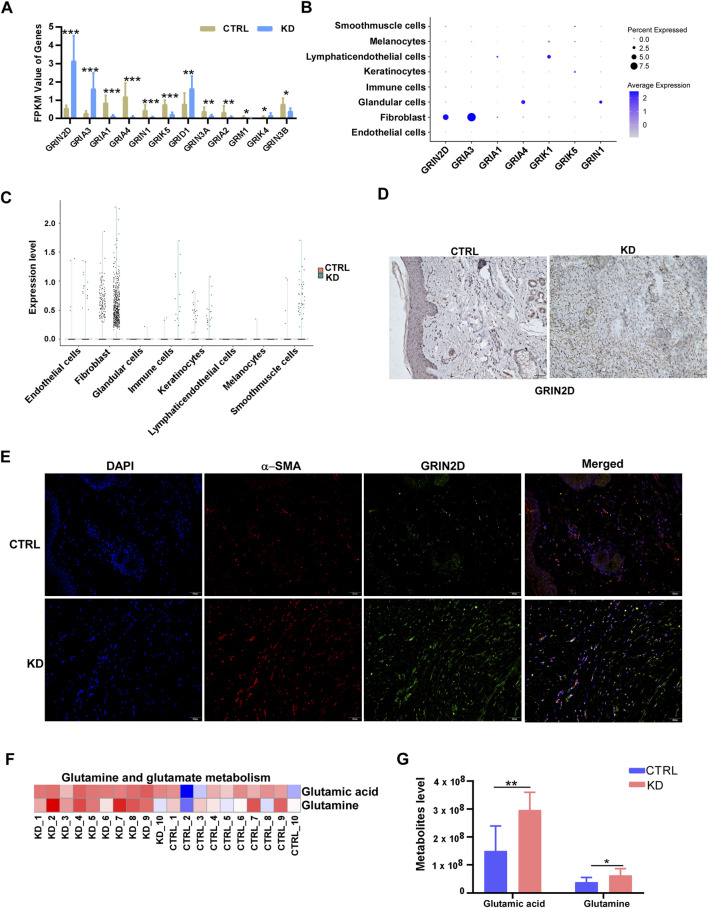
Glutamate metabolic and glutamate receptor (GRIN2D) is increased in keloids and fibroblasts. **(A)** FPKM values of glutamate receptor genes (GRIN2D, GRIA1, GRIA2, GRIK1, GRIK2, GRIK5, GRIN1, GRIM1, GRIMX, GRIN1B) in keloid compared to normal skin controls. **(B)** Dot plot of single-cell RNA-seq data depicting the distribution of GRIN2D and related glutamate receptor genes across major skin cell populations. **(C)** Expression levels of GRIN2D across different cell types in CTRL and KD skin, showing preferential upregulation in fibroblasts from KD lesions. **(D)** Representative immunohistochemical staining for GRIN2D in CTRL and KD tissues, demonstrating stronger GRIN2D signal in keloid dermis. **(E)** Immunofluorescence of α-SMA and GRIN2D in keloid and normal skin tissues. **(F,G)** Heatmap **(F)** and bar graph **(G)** of targeted metabolomics for glutamic acid and glutamine in individual CTRL and KD samples, illustrating a shift in glutamine–glutamate metabolism in KD. p-values were determined by one-way ANOVA: *p < 0.05, **p < 0.01, ***p < 0.001.

To investigate whether the glutamate metabolic regulates keloid process, we analyzed the expression of glutamate receptors in keloid and healthy skin tissues. RNA-seq data revealed that *GRIN2D* was significantly upregulated in keloids compared to healthy controls, with other receptors like *GRIA1* and *GRID1* also showing notable expression changes (Figure 3A). Immunohistochemical staining of GRIN2D shown that the keloid samples exhibit stronger staining intensity compared to controls (Figure 3D), confirming the elevated expression of this receptor in keloid tissue.

To assess GRIN2D expression at the single-cell level, we analysis the scRNA-seq data from GEO database (GSE163973), which indicated that *GRIN2D* is predominantly expressed in fibroblasts (Figures 3B,C). Confocal microscopy images showed co-localization of GRIN2D with α-SMA, an activated fibroblast marker, which confirmed that fibroblasts express GRIN2D at high levels, suggesting its involvement in fibroblast activation and tissue remodeling processes (Figure 3E). Additionally, our untargeted metabolic assay (Supplementary Figure S1A–S1C) revealed significant increases in glutamic acid and glutamine metabolic pathways in keloid tissues compared to controls (Figures 3F,G), highlighting the metabolic shifts associated with keloid formation.

Overall, these findings indicate that the expression of glutamate receptors, particularly GRIN2D, is markedly elevated in keloid tissues. This elevation correlates with alterations in glutamate metabolism, suggesting a potential role for GRIN2D in the pathogenesis of keloids.

### 3.4 Glutamate reprogram keloid fibroblasts transcripts

**FIGURE 4:**
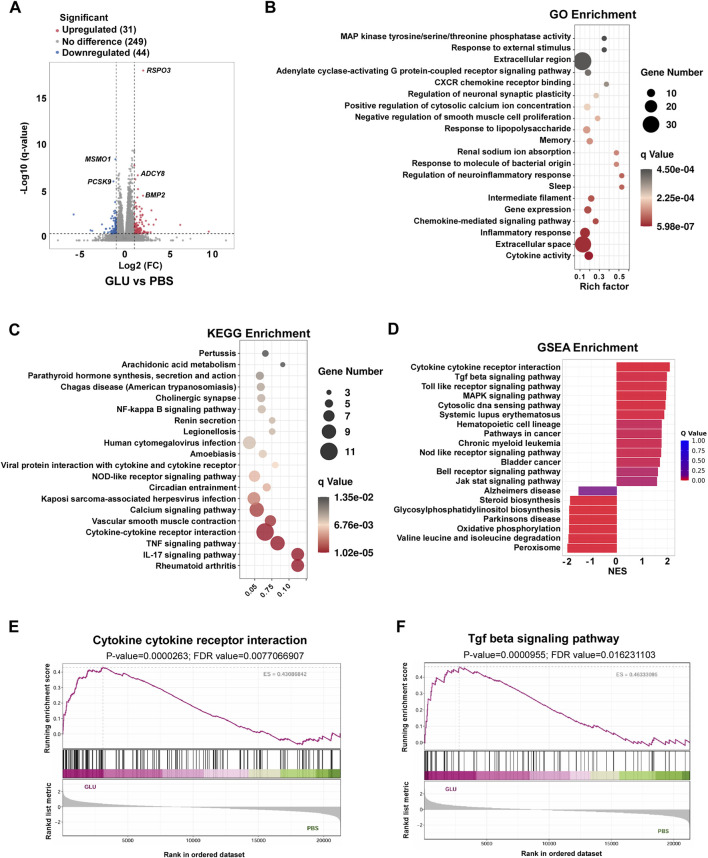
Glutamate reprogram keloid fibroblasts transcripts. **(A)** Volcano plot show DEGs in HKF cells upon 100 μM glutamate stimulate for 24 h. **(B,C)** GO enrichment of DEGs **(B)** with bubble size representing the number of genes in each term and color indicating the adjusted q value, and KEGG enrichment of DEGs showing the top enriched signaling pathways related to cytokine and immune responses **(C)**. **(D)** Barplot of GSEA enrichment of DEGs, resented as normalized enrichment scores (NES); bars to the right indicate pathways positively enriched in GLU compared with PBS. **(E,F)** GSEA enrichment of cytokine-cytokine receptor interaction **(E)** and TGF beta signaling pathway **(F)**.

To further analyze the effects of glutamate and glutamine metabolism on keloid disease, we stimulated keloid fibroblasts *in vitro* with glutamate and subsequently performed RNA-seq. The results revealed substantial alterations in gene expression profiles, specifically, 103 genes were found to be significantly upregulated, while 68 genes were downregulated in fibroblasts stimulated with glutamate (Figure 4A). Notably, several genes, including *BMP2*, *ASCY8*, *MSMO1*, and *PCSK9*, an essential regulator of cholesterol biosynthesis, exhibited significant changes in expression upon glutamate stimulation (Figure 4A). GO and KEGG enrichment analysis found several immune related pathways were enriched in glutamate stimulated fibroblasts, including CXCR chemokine receptor pathway, chemokine mediated signaling pathway, cytokine-cytokine receptor interaction and TNF signaling pathway (Figures 4B,C). Additionally, GSEA analysis confirmed cytokine-cytokine receptor interaction and TGF beta signaling pathway were upregulated in glutamate stimulated fibroblasts cells (Figures 4D–F). These data together suggest that glutamate significantly influences proinflammatory responses in keloid fibroblasts, indicating a potential role in keloid pathogenesis.

### 3.5 Glutamate promoted inflammatory responses in keloid fibroblasts

**FIGURE 5:**
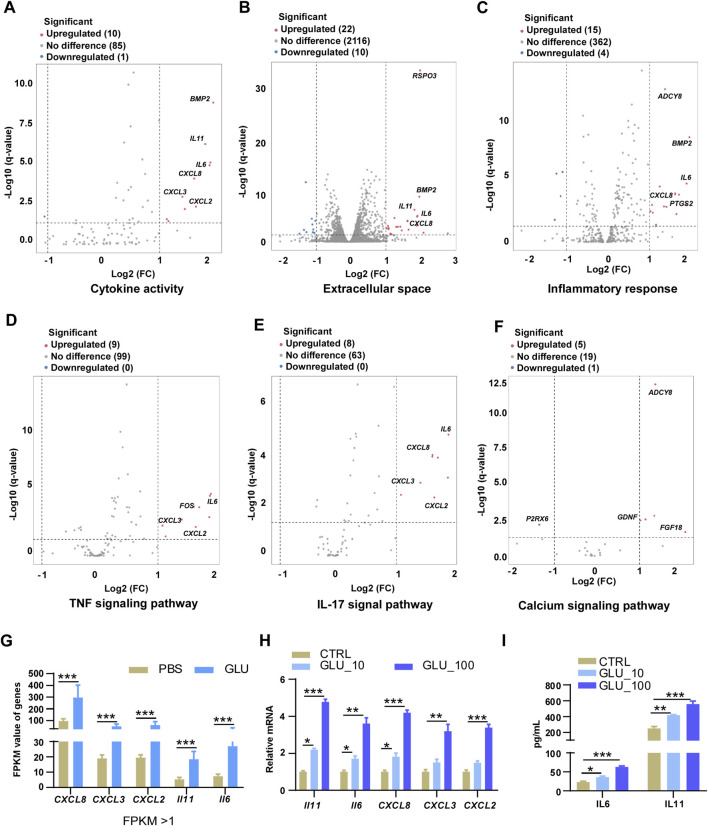
Glutamate promotes inflammatory responses in keloid fibroblasts. **(A–C)** Volcano plot show DEGs that associated to cytokine activity pathway **(A)**, extracellular space pathway **(B)**, and inflammatory response pathway **(C)** in HKF cells upon 100 μM glutamate stimulate for 24 h. **(D–F)** Volcano plot and heatmap show DEGs that associated to TNF signaling pathway **(D)**, IL-17 signaling pathway **(E)**, and calcium signaling pathway **(F)** in HKF cells upon 100 μM glutamate stimulate for 24 h. **(G)** FPKM of inflammatory genes (CXCL8, CXCL3, CXCL2, IL11, IL6) of RNA-seq in keloid and normal tissues in HKF cells upon 100 μM glutamate stimulate for 24 h. **(H)** RT-PCR of inflammatory genes in HKF cells upon 10 μM or 100 μM glutamate stimulate for 24 h. **(I)** ELISA of IL-6 andIL-11 in HKF cells upon 10 μM or 100 μM glutamate stimulate for 24 h p-values were determined by one-way ANOVA: *p < 0.05, **p < 0.01, ***p < 0.001.

Given that glutamate promoted inflammatory responses in fibroblasts, we aimed to elucidate the mechanisms by which glutamate enhances inflammation in these cells. Our RNA-seq results revealed significant upregulation of genes associated with cytokine activity, extracellular space, and inflammatory response pathways following glutamate stimulation (Figures 5A–C). Notably, inflammatory genes such as *CXCL2*, *CXCL3*, *CXCL8*, *IL11*, and *IL6* were enriched within these pathways (Figures 5A–C), indicating a robust inflammatory response upon glutamate stimulation.

Further analysis revealed alterations in key inflammatory signaling pathways, including the TNF signaling pathway and the IL-17 signaling pathway. Genes like *CXCL2*, *CXCL3*, *IL6*, and *TNFAIP3* exhibited significant upregulation in fibroblasts stimulated by glutamate (Figures 5D–F). Additionally, we observed upregulation in the calcium signaling pathway, with genes such as *ADCY8* and *SLC8A2* showing notable increases upon glutamate stimulation (Figure 5F). To validate our RNA-seq findings, we performed qPCR, which confirmed the upregulation of pro-inflammatory factors (Figures 5G,H). ELISA assays further supported these results, demonstrating increased IL-6 and IL-11 levels of inflammatory cytokines (Figure 5I).

Overall, these findings underscored the role of glutamate in enhancing inflammatory responses in fibroblasts, which may contribute to the development and progression of keloid disease.

## 4 Discussion

In this study, we demonstrated a pronounced upregulation of the glutamate receptor subunit GRIN2D in fibroblasts within keloid tissue, accompanied by enhanced glutamine–glutamate metabolism. Functionally, glutamate stimulation markedly amplified the production of inflammatory mediators, particularly the cytokines IL-6 and IL-11 and the chemokines CXCL2, CXCL3, and CXCL8. Together, these findings indicated that fibroblast-intrinsic glutamate signaling, driven by glutamine metabolism and mediated at least in part through GRIN2D, critically reinforces the proinflammatory phenotype of keloid fibroblasts. Thus, our work implicated the glutamine–glutamate–GRIN2D axis as a key contributor to keloid progression and highlights it as a promising metabolic and signaling target for therapeutic intervention.

Glutamine serves as a key anaplerotic substrate in proliferating and activated fibroblasts, fueling the tricarboxylic acid cycle, supporting biosynthesis and redox homeostasis, and facilitating collagen production and myofibroblast differentiation (Gibb et al., 2022). Its conversion to glutamate through glutaminase not only sustains intracellular metabolism but also increases the pool of glutamate available for export and receptor-mediated signaling (Zhang et al., 2024). Extracellular glutamate, in turn, can activate ionotropic receptors such as NMDA receptors (including GRIN2D-containing channels) and metabotropic glutamate receptors, leading to calcium influx and the activation of downstream pathways such as NF-κB, MAPK, and calcineurin–NFAT (Del Arroyo et al., 2019; Vieira et al., 2020). In fibroblasts, NF-κB and MAPK have been implicated in promoting proliferation, survival, ECM deposition, and cytokine and chemokine production (Wang et al., 2024). Consistent with this, our data showed that enhanced glutamine–glutamate metabolism in keloid tissue coincides with GRIN2D upregulation and glutamate-induced transcriptional reprogramming of keloid fibroblasts toward a highly inflammatory, potentially profibrotic state. These observations supported a model in which metabolic rewiring of glutamine and glutamate not only meets the bioenergetic and biosynthetic demands of keloid fibroblasts but also actively shapes their signaling landscape to sustain chronic inflammation and fibrosis.

IL-6 and IL-11, two closely related gp130-family cytokines, have emerged as key mediators at the interface of inflammation and fibrosis, providing a mechanistic link that is highly relevant to keloid pathogenesis (Heichler et al., 2020). Both cytokines signal predominantly through JAK/STAT3 and MAPK pathways to promote fibroblast activation, survival, and extracellular matrix production (Zhuang et al., 2019; Zuo et al., 2025). In multiple fibrotic diseases—including pulmonary, cardiac, hepatic, and dermal fibrosis—IL-6 has been shown to enhance myofibroblast differentiation, upregulate collagen and other ECM components, and sustain a chronic inflammatory milieu through paracrine and autocrine loops (Li et al., 2022). IL-11, once considered mainly a hematopoietic cytokine, is now recognized as a potent profibrotic factor that directly drives fibroblast proliferation, collagen synthesis, and tissue stiffening, often downstream of canonical profibrotic pathways such as TGF-β (Zhong et al., 2025). Consistent with these observations, our data showed that glutamate stimulation of keloid fibroblasts markedly increases IL-6 and IL-11 expression and secretion, suggesting that these cytokines may act as critical effectors of glutamate-driven fibroblast reprogramming. We therefore proposed that IL-6 and IL-11 not only amplify local inflammation but also reinforce the fibrotic phenotype of keloid fibroblasts, potentially establishing self-sustaining cytokine–fibroblast feedback circuits.

CXCL2, CXCL3, and CXCL8 are ELR^+^ CXC chemokines that primarily signal through CXCR2 (with CXCL8 also engaging CXCR1) to orchestrate the recruitment and activation of neutrophils and other inflammatory cells at sites of tissue injury (Fernandez-Avila et al., 2023). Beyond their canonical roles in acute inflammation, accumulating evidence implicates these chemokines as important modulators of chronic inflammation and fibrosis in organs such as the lung, liver, kidney, and skin, where their elevated expression correlates with disease severity and extracellular matrix accumulation (Dunbar et al., 2023; Nie et al., 2023). Mechanistically, CXCL2/3/8 not only drive sustained influx of neutrophils capable of releasing proteases, reactive oxygen species, and profibrotic mediators (e.g., TGF-β, IL-1β), but may also act directly on CXCR1/2-expressing fibroblasts to promote proliferation, survival, myofibroblast differentiation, and collagen production, in part through activation of NF-κB, MAPK, and JAK–STAT pathways (Santolla et al., 2022). In this context, our observation that glutamate stimulation of keloid fibroblasts markedly induced CXCL2, CXCL3, and CXCL8 suggests that glutamine–glutamate metabolic reprogramming may reinforce a chemokine-driven inflammatory circuit that sustains leukocyte recruitment and fibroblast activation within keloid lesions.

This study has several limitations. First, our findings were primarily based on *in vitro* experiments and multi-omics analyses, and we lacked systematic *in vivo* validation in appropriate animal models or longitudinal clinical cohorts; therefore, the dynamic role of the glutamate–GRIN2D axis in scar initiation, progression, and resolution *in vivo* remains to be fully elucidated. Second, although we identified GRIN2D as a potentially critical mediator at the gene and protein levels, we had not yet comprehensively evaluated the therapeutic efficacy and safety of targeting GRIN2D using specific inhibitors *in vivo* or in models that more closely mimic the clinical context. Future studies employing suitable fibrosis/scar models, combined with pharmacologic GRIN2D inhibition or genetic manipulation, will be essential to define its anti-fibrotic potential and translational feasibility.

## 5 Conclusion

In conclusion, our study identifies fibroblast-intrinsic glutamate signaling, driven by enhanced glutamine–glutamate metabolism and GRIN2D upregulation, as a key amplifier of IL-6/IL-11 and CXCL2/3/8 production and a promising metabolic–signaling target for therapeutic intervention in keloid disease.

## Data Availability

The RNA-seq data and the metabolomics data were uploaded in the National Genomics Data Center database under accession number PRJCA053133, and PRJCA053129.
