# Supplementary material for: Glutamate enhances the production of inflammatory cytokines IL-6 and IL-11, as well as chemokines CXCL2, CXCL3, and CXCL8 in keloid fibroblasts
Source: Front Mol Biosci. 2026 Jan 5;12:1720876. doi: 10.3389/fmolb.2025.1720876 (PMC12812582; doi:10.3389/fmolb.2025.1720876)
Supplement: Supplementary file 1 [file Image1.pdf]

Supplementary figure 1. Untargeted metabolomics analysis of keloid and normal control

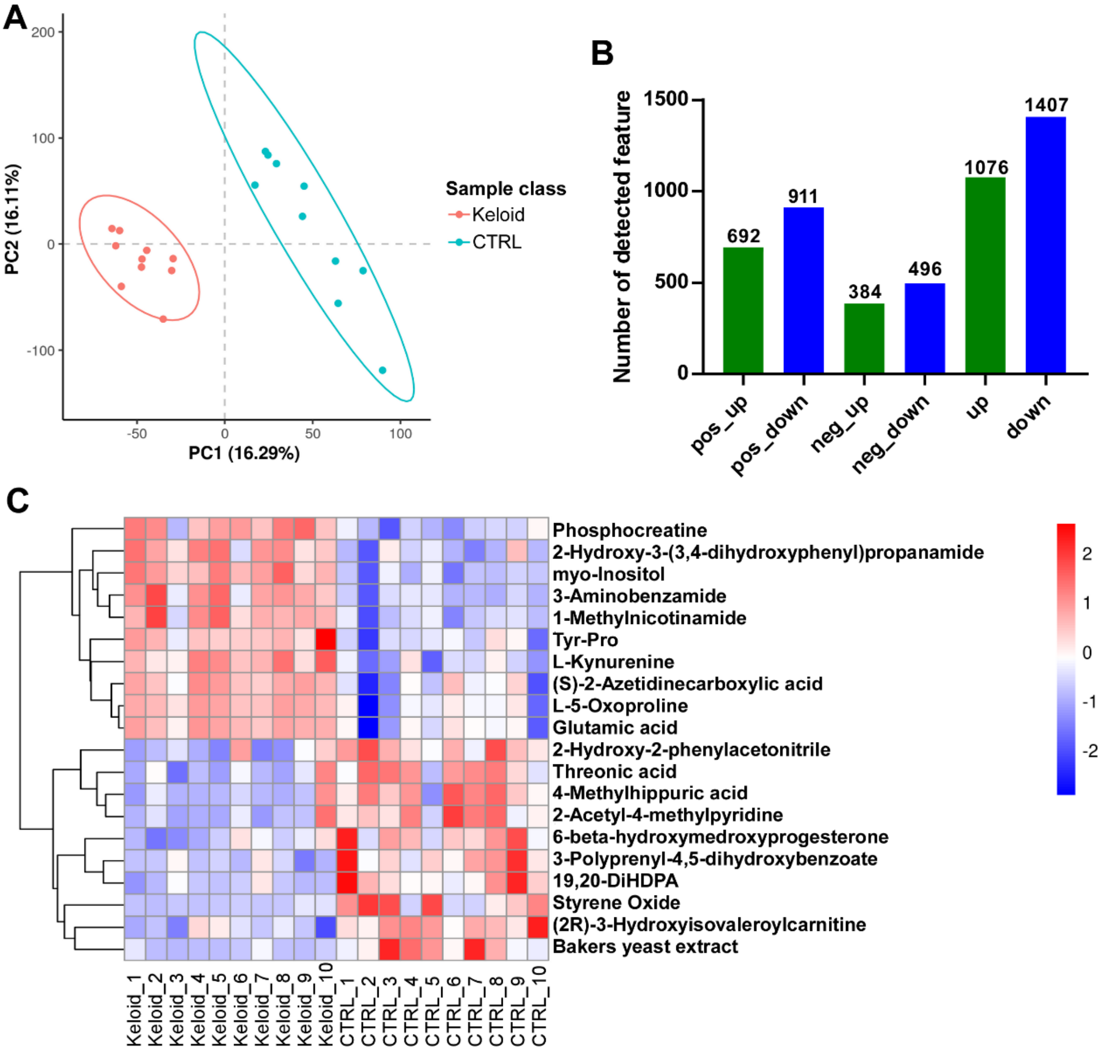

(A) Principal component analysis (PCA) revealed two relatively independent clusters along PC1 and PC2, corresponding to keloid and control samples, respectively.

(B) Differential feature statistics further demonstrated extensive metabolic alterations in both positive and negative ion modes.

(C) Hierarchical clustering of differential metabolites, visualized as a heatmap, showed systematic changes in the levels of multiple key metabolites between keloid and control samples.
